# p21-Activated Kinase (PAK) Regulates Cytoskeletal Reorganization and Directional Migration in Human Neutrophils

**DOI:** 10.1371/journal.pone.0073063

**Published:** 2013-09-03

**Authors:** Asako Itakura, Joseph E. Aslan, Branden T. Kusanto, Kevin G. Phillips, Juliana E. Porter, Paul K. Newton, Xiaolin Nan, Robert H. Insall, Jonathan Chernoff, Owen J. T. McCarty

**Affiliations:** 1 Department of Cell and Developmental Biology, Oregon Health and Science University, Portland, Oregon, United States of America; 2 Department of Biomedical Engineering, Oregon Health and Science University, Portland, Oregon, United States of America; 3 Division of Hematology and Medical Oncology, School of Medicine, Oregon Health and Science University, Portland, Oregon, United States of America; 4 Department of Aerospace and Mechanical Engineering, University of Southern California, Los Angeles, California, United States of America; 5 CRUK Beatson Institute for Cancer Research, Glasgow, United Kingdom; 6 Fox Chase Cancer Center, Philadelphia, Pennsylvania, United States of America; University of Birmingham, United Kingdom

## Abstract

Neutrophils serve as a first line of defense in innate immunity owing in part to their ability to rapidly migrate towards chemotactic factors derived from invading pathogens. As a migratory function, neutrophil chemotaxis is regulated by the Rho family of small GTPases. However, the mechanisms by which Rho GTPases orchestrate cytoskeletal dynamics in migrating neutrophils remain ill-defined. In this study, we characterized the role of p21-activated kinase (PAK) downstream of Rho GTPases in cytoskeletal remodeling and chemotactic processes of human neutrophils. We found that PAK activation occurred upon stimulation of neutrophils with f-Met-Leu-Phe (fMLP), and PAK accumulated at the actin-rich leading edge of stimulated neutrophils, suggesting a role for PAK in Rac-dependent actin remodeling. Treatment with the pharmacological PAK inhibitor, PF3758309, abrogated the integrity of RhoA-mediated actomyosin contractility and surface adhesion. Moreover, inhibition of PAK activity impaired neutrophil morphological polarization and directional migration under a gradient of fMLP, and was associated with dysregulated Ca^2+^ signaling. These results suggest that PAK serves as an important effector of Rho-family GTPases in neutrophil cytoskeletal reorganization, and plays a key role in driving efficient directional migration of human neutrophils.

## Introduction

Chemotaxis, the directed migration of cells driven by a gradient of external factors, is critical for the initial phases of innate immunity in which neutrophils sense chemoattractant mediators and migrate from the circulation through the endothelium to combat invading pathogens at sites of infection [1]. During chemotaxis, neutrophils undergo dramatic morphological changes as lamellipodia at the leading edge extend toward chemoattractant sources and a trailing edge forms at the neutrophil rear, termed the uropod. These polarized structures support the efficient migration of neutrophils at speeds of up to 30 µm/min [2]. Although many studies have described the molecular mechanisms that underlie the steps of chemotaxis (such as directional sensing, polarization and motility), the manner in which neutrophil ‘frontness’ vs. ‘backness’ signals are coordinated to direct migration is still not fully understood.

Most chemoattractants, including the bacteria-derived chemotactic peptide f-Met-Leu-Phe (fMLP), bind to G protein-coupled receptors (GPCRs) expressed on the neutrophil surface. GPCR-ligand binding activates heterotrimeric G proteins and triggers various intracellular signaling pathways. The Rho family of GTPases, including Rac, Cdc42 and RhoA, have been shown to play key roles in the spatial and temporal regulation of neutrophil cytoskeletal remodeling downstream of chemoattractant receptors during chemotaxis [2]. In their activated forms, Rac and Cdc42 promote the extension and stabilization of an actin-rich leading edge at the front of neutrophils to generate a motile force, while active RhoA controls myosin II-dependent contractility and uropod retraction. Many signaling pathways have been shown to participate in a feedback loop that maintains the formation of a single leading edge and uropod. In the context of cytoskeletal rearrangement, the family of p21-activated kinases (PAKs) is a well-characterized target of Rac and Cdc42. To date, six isoforms of PAKs have been identified; PAK1, 2, 3 (Group I PAKs) and PAK 4, 5, 6 (Group II PAKs). Group I and II PAKs differ in their structural organizations and biochemical features including activation mechanisms [3]. The binding of Rac or Cdc42 GTPases to the p21-binding domain (PBD) of group I PAKs induces autophosphorylation and activation of PAK as serine/threonine kinases, whereas the binding of Cdc42 to PBD does not serve to activate group II PAKs [3]. In neutrophils, rapid phosphorylation of PAK1 and PAK2 isoforms has been observed after treatment with various agonists [4], [5], and PAK1 has been found at the leading edge and phagocytic cup [6]. In a study using mouse neutrophils as well as non-myeloid (e.g. COS-7) and myeloid cell lines (e.g. HL-60 and RAW274), PAK1 induced Cdc42 activation by forming a complex with Gβγ and the guanine-nucleotide exchange factor (GEF) PIXα to promote actin polymerization and regulate PTEN distribution for efficient directional sensing [7]. However, the characterization of PAK function in human neutrophils has been hindered by the technical limitation that neutrophils are not susceptible to genetic manipulation *in vitro*, as they are terminally differentiated and have a short life span. Accordingly, studies of the functional roles of PAK in human neutrophils have been restricted to the use of gene transfection/knockdown strategies in leukemic cell lines. While PAK1- and PAK2-knockout mice have recently been established [8], [9], [10], [11], [12], [13], [14], the neutrophil phenotype in these mice has not yet been described.

In this study, we characterized the roles of the PAK signaling in relation to PI3K and Rho GTPase systems during fMLP-driven cytoskeletal reorganization in primary human neutrophils. Our data suggest that PAK2 is activated and accumulates to the neutrophil leading edge in response to fMLP to support Rac/Cdc42-mediated actin dynamics in a localized manner. In addition, PAK inhibition altered the subcellular localization of active RhoA and induced aberrant formation of vinculin-rich complexes. PAK kinase activity played a critical role in chemotaxis of human neutrophils as PAK inhibition led to a loss of directionality, increased spreading and decreased migration speed, whereas Rac or PI3K inhibition resulted in impaired directionality or polarization, respectively. Taken together, these results suggest that PAKs establish a ‘frontness’ signal by negatively regulating the surface adhesion and Rho-dependent ‘backness’ signals in human neutrophils, thus providing a mechanism for the crosstalk between Rho-family GTPases in neutrophil cytoskeletal dynamics and cell migration.

## Materials and Methods

### Reagents

For immunohistochemistry and western blot experiments, anti-αPAK (PAK1; sc-882), γPAK (PAK2; sc-373740), βPAK (PAK3; sc-1871) and PAK4 (sc-28779) were purchased from Santa Cruz (Dallas, TX, USA). Anti-PAK1/2/3 pThr423 (44–942G) was from Invitrogen (Grand Island, NY, USA). Anti-PAK2 pSer20 (2607) and myosin light chain2 pSer19 (3675) were from Cell Signaling (Boston, MA, USA). Anti-Rac1 (23A8) and Y27632 was from Millipore (Billerica, MA, USA). Anti-active Rac-GTP (26903), active RhoA-GTP (26904) and active-Cdc42-GTP (26905) antibodies were from NewEast Biosciences (King of Prussia, PA, USA). PF3758309 was prepared as previously described [12], [15], [16]. All other reagents were from Sigma-Aldrich (St. Louis, MO, USA) or previously named sources [16], [17].

### Ethics Statement

All human blood donors provided written informed consent in accordance with a protocol approved by the Oregon Health & Science University Institutional Review Board.

### Preparation of Human Neutrophils

Human neutrophils were purified as previously described [18]. Briefly, human venous blood was drawn from healthy donors into citrate-phosphate-dextrose and layered over an equal volume of Polymorphprep, followed by centrifugation at 500 g for 45 min at 18°C. The lower layer containing neutrophils was collected and washed with Hank’s balanced salt solution (HBSS) by centrifugation at 400 g for 10 min. To remove contaminating red blood cells, the pellet was resuspended in sterile H_2_O for 30 sec, followed by the immediate addition of 10× PIPES buffer (250 mM PIPES, 1.1 mM NaCl, 50 mM KCl; pH 7.4). After centrifugation at 400 g for 10 min, the pellet was resuspended in PMN buffer [HBSS containing 2 mM CaCl_2_, 2 mM MgCl_2_ and 1% w/v bovine serum albumin (BSA)].

### Western Blotting

After indicated treatments, human neutrophils were lysed in protein extraction reagent (#78505; Thermo Scientific, Waltham, MA, USA). Lysates were separated by SDS-PAGE and transferred onto PVDF membranes. The membranes were blocked with 5% BSA for 1 hr prior to incubation with primary antibodies overnight at 4°C. Following washing with TBS-T, membranes were incubated with the appropriate secondary antibodies. Subsequent immunoblotting was carried out as previously described [17], [19].

### Immunofluorescence and Total Internal Reflection Fluorescence (TIRF) Microscopy

Purified human neutrophils (2×10^6^/ ml) were incubated on fibronectin-coated surfaces at 37°C for 1 hr in the presence of inhibitors or vehicle (DMSO), followed by stimulation with fMLP. After fixation with 4% paraformaldehyde for 5 min, cells were permeabilized with 80% acetone for 3 min and blocked with blocking buffer (10% fetal bovine serum, 5 mg/ml BSA in PBS) for 10 min. For phospho-PAK staining, cells were permeabilized with methanol. Neutrophils were stained with anti-PAK1/2/3 pThr423 (1∶100), anti-PAK2 pSer20 (1∶50), anti-Rac (1∶100), anti-active Rac-GTP (1∶100), anti-active RhoA-GTP (1∶200), anti-active Cdc42-GTP (1∶100) anti-myosin light chain2 pSer19 (1∶200), anti-vinculin (1∶100), or anti-actin (1∶100) in blocking buffer overnight at 4°C. Secondary antibodies conjugated with AlexaFluor 488 or AlexaFluor 546 (1∶500) and/or TRITC-phalloidin (1∶1000) in blocking buffer were added and incubated for 2 hr in dark. Coverslips were mounted onto glass slides and visualized with a Zeiss Axiovert fluorescent microscope. For TIRF microscopy, purified neutrophils were plated in 8-well chamber slide (Nunc) and treated with vehicle or inhibitors followed by stimulation with fMLP. Cells were fixed, permeabilized and stained for vinculin as described above. Vinculin immunofluorescence in a focal section of the neutrophil within ∼150 nm from the surface of coverslip was excited with a 488 nm-laser and detected via TIRF microscopy using a Nikon TE300 microscope equipped with a Nikon 60× oil immersion objective (NA = 1.49) and an electron multiplied CCD camera. For data presentation, fluorescent intensities of each image were adjusted based on signals detected in neutrophil samples treated without primary antibody. Image analyses were performed using Colocalization Finder of ImageJ, or a custom program in MATLAB (The Mathworks, Inc., Natick, MA, USA).

### Insall Migration Chamber Assays

Neutrophil chemotaxis was recorded as previously described [18]. Briefly, purified human neutrophils were incubated at 37°C for 30 min on fibronectin-coated coverslips. The coverslips with adherent cells were then inverted onto an Insall chamber. After PMN buffer in the outer wells was aspirated, fMLP-containing PMN buffer was added to induce chemotaxis. Images were obtained every 10 s for 30 min at 40×using differential interference contrast (DIC) microscopy as previously described [18]. In selected experiments, cells were pretreated with indicated inhibitors for 15 min at room temperature prior to the experiment. Images were analyzed using Image J plugin MtrackJ and quantified using the CircStat toolbox for MATLAB.

### Intracellular Ca^2+^ Measurement

Human neutrophils (2×10^6/ ^ml) were loaded with fluo-4 AM (final 2 µM) and plated on fibronectin-surface for 30 min at 37°C. After washing with Ca^2+^-free HBSS, cells were incubated for another 30 min at 37°C in PMN buffer and inverted onto an Insall chamber. Intracellular Ca^2+^ spikes in neutrophils were monitored for 15 min at 40×using a Zeiss Axiovert inverted fluorescent microscopy and analyzed using a custom MATLAB program.

### Analysis of Data

Data are shown as means±SEM. For the quantification of immunofluorescence images, the Jarque-Bera test was used to evaluate normality of all parameters. One-way analysis of variance with Bonferonni *post hoc* correction was used to assess statistical significance among parameters across multiple normally distributed cell parameters. The Kruskal-Wallis test was used to assess significance among non-normally distributed parameters. For the migration speed analysis, statistical analysis was performed using Student’s t test. P-values of 0.05 or less were considered significant.

## Results

### PAK2 Translocates to Leading Edge upon fMLP Stimulation

**Figure 1:**
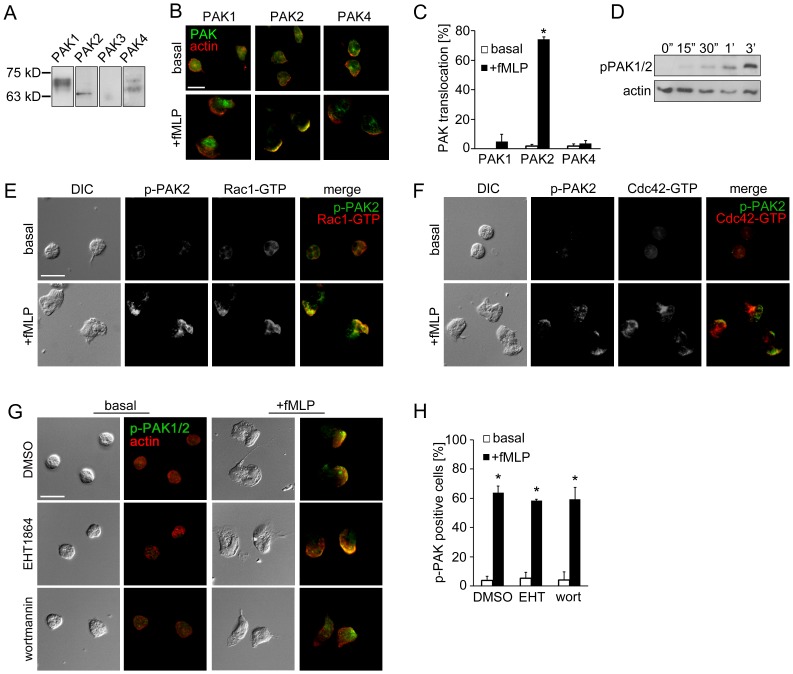
PAK2 localizes to the leading edge of activated neutrophils. Replicate samples of total neutrophil (PMN) cell lysates (50 µg per lane) were analyzed for (A) the expression of PAK1 (68 kD), PAK2 (61 kD), PAK3 (65 kD) and PAK4 (72 kD) or (D) phosphorylation of PAK1/2 Thr432/402 at indicated time points after the addition of fMLP (10 nM) by Western blot. Human neutrophils adherent on fibronectin surfaces were treated in the presence or absence of fMLP (10 nM) for 3 min and stained for (B) PAK1, PAK2 or PAK4 (green) and F-actin (red), (E) phospho-PAK2 Ser20 (green) and Rac1-GTP (red), (F) phospho-PAK2 Ser20 (green) and Cdc42-GTP (red), or (G) phospho-PAK1/2 Thr423/402 (green) and F-actin (red). In selected experiments, neutrophils were pretreated with vehicle (0.1% DMSO), EHT1864 (Rac1/2 inhibitor, 50 µM) or wortmannin (PI3K inhibitor, 100 nM). Results are quantified from at least 60 cells and presented as the mean percentage±SEM of (C) neutrophils displaying PAK immunofluorescence at actin-rich leading edge, or (H) neutrophils displaying phospho-PAK1/2 immunofluorescence in the fields of view. Representative images obtained from 3 independent experiments are shown. * *P<0.05,* compared to the basal level. Scale ba = 10 µm.

The PAK isoforms described in humans (PAK1–6) display a wide range of tissue distribution. PAK2 and PAK4 are ubiquitously expressed, while PAK1, PAK3, PAK5 and PAK6 expression are specific for tissues such as brain (PAK1, PAK3, PAK5 and PAK6) and spleen (PAK1) [20], [21]. Previous studies of the PAK pathways in neutrophils have relied on biochemical assays to monitor PAK1 and PAK2 activation [4], [5]. However, little is known about the expression and activity of PAK isoforms in regulating neutrophil cytoskeletal reorganization and migration. To characterize the expression of PAK isoforms in human neutrophils, whole human neutrophil lysates were separated by SDS-PAGE and examined for PAK expression by Western blot using PAK isoform-specific antibodies. As shown in Fig. 1A, neutrophils express detectable levels of PAK1, PAK2 and PAK4. Next, to examine PAK isoform expression and subcellular localization, PAKs were studied by immunofluorescence microscopy of fixed neutrophils. PAK1, PAK2 and PAK4 were all detected in the cytosol of unstimulated neutrophils (Fig. 1B). Upon the stimulation with the bacteria-derived peptide fMLP, PAK2 colocalized with the actin-rich leading edge of neutrophils, whereas PAK1 and PAK4 localization was excluded from leading edge and primarily found in the cytosol and/or the back of stimulated cells (Fig. 1B and 1C).

We next examined whether the translocation of PAK2 to the leading edge is associated with kinase activation downstream of fMLP receptor signaling. In primary human neutrophils, PAK undergoes rapid phosphorylation at specific Ser and Thr residues in response to a number of stimuli [4], [5]. Phosphorylation of PAK1 Thr423 or PAK2 Thr402 is required for full catalytic function [22], [23], whereas phosphorylation at Ser20 of PAK2 regulates binding to the adaptor protein Nck to control PAK membrane localization as well as PAK kinase activity [24], [25]. The PAKs are activated upon the binding of the ∼21 kD Rho GTPases Rac and Cdc42 [24], [25]. Along these lines, Rac and Cdc42 GTPases promote lamellipodia formation and regulate lamellipodia stability through a positive feedback loop at the actin-rich leading edge of migrating cells [26], [27], including neutrophils [2], [28], [29]. Our Western blot analysis showed that PAK1 Thr423 and PAK2 Thr402 residues were phosphorylated within 3 min after neutrophil stimulation with fMLP (Fig. 1D). In parallel, phosphorylated PAK Ser20 accumulated at the neutrophil leading edge and colocalized with active, GTP-bound Rac1 (71.7±4.4% colocalization; Fig. 1E) and Cdc42 (49.8±6.0% colocalization; Fig. 1F). In parallel, Rac2, a Rac isoform expressed in myeloid cells, was found to localize to the cytosol and toward the leading edge with 63.6±8.4% of the immunofluorescence signal of Rac2 colocalizing with Rac1, following neutrophil stimulation with fMLP for 3 min. Together, these results suggest that a coupled activation of Rac/Cdc42 and PAK2 are involved in leading edge formation during neutrophil migration.

Next, we investigated the mechanisms that regulate PAK activation in lamellipodia dynamics of activated neutrophils. To detect the activation of PAK, we performed immunofluorescence microscopy experiments using primary antibodies specific for phosphorylated PAK1/2 Thr423/402. Fluorescence microscopy revealed Threonine-phosphorylated PAK1/2 in neutrophils upon stimulation with fMLP, which localized to the actin-rich leading edge (Fig. 1G). To test the role of Rac GTPase in neutrophil PAK activation, we next analyzed the effect of Rac inhibition on PAK activation and neutrophil morphology using the Rac1/2 inhibitor EHT1864. In Rac-inhibited neutrophils, phosphorylated PAK1/2 Thr423/402 remained at the leading edge (Fig. 1G and 1H), while phosphorylation at PAK2 Ser20 was undetectable (data not shown), suggesting that Rac-independent and-dependent mechanisms are required for full PAK activation and PAK function in neutrophils.

In neutrophils, Rac activation is in part regulated by PI3Ks which promote the localization of Rac-activating GEFs to the neutrophil leading edge [30], [31], [32]. Accordingly, an impairment of PAK2 activation has been reported downstream of RANTES-induced chemotaxis signaling in PI3Kγ-deficient mouse macrophages [33]. As shown in Fig. 1G and 1H, treatment of neutrophils with the PI3K inhibitor, wortmannin, failed to block PAK phosphorylation in response to fMLP, but led to a non-polarized accumulation of actin and phospho-PAK. This result suggests that like Rac, PI3K activity may also play a role in the intracellular trafficking of active PAK, but not directly in PAK activation.

### PAK Activation and Rho GTPases Mediate Neutrophil Backness Signals

**Figure 2:**
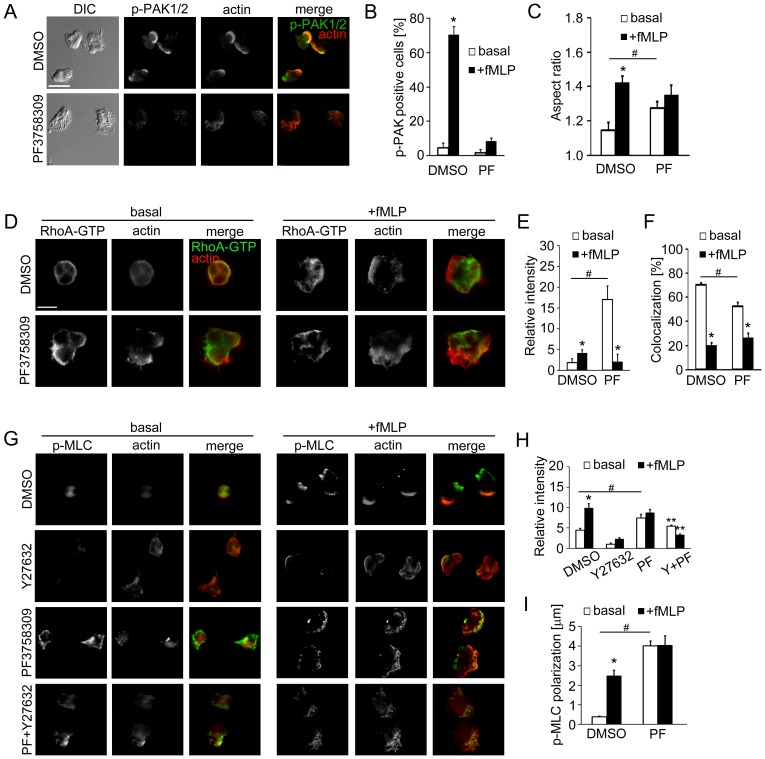
PAK inhibition leads to an accumulation of active RhoA and phosphorylated myosin light chain. (A) Human neutrophils adherent on fibronectin surfaces were pretreated with vehicle (0.1% DMSO) or PF3758309 (PF; PAK inhibitor, 10 µM), and stimulated with fMLP (10 nM) for 3 min and stained for phospho-PAK1/2 Thr423/402 (green) and F-actin (red). (B) The mean percentage of cells displaying phospho-PAK1/2 immunofluorescence at basal level (white bars) or after stimulation (black bars) was quantified from at least 50 cells per treatment. (C) Cell circularity was analyzed using MATLAB and presented as aspect ratio of individual neutrophils at basal level (white bars) or after fMLP stimulation (black bars). (D) Neutrophils were pretreated as indicated in (A) and stimulated with fMLP (10 nM). Cells were stained for active RhoA-GTP (green) and F-actin (red). (E) The mean relative intensity of RhoA-GTP immunofluorescence or (F) the mean percentage of RhoA-GTP/actin colocalization of individual cell area at basal level (white bars) or after fMLP stimulation (black bars). (G) Neutrophils were pretreated with the inhibitors indicated in (A), or with Y27632 (ROCK inhibitor, 10 µM), or a combination of 10 µM PF3758309 and 10 µM Y27632 (PF+Y27632) and stained for phospho-myosin light chain (p-MLC; green) and F-actin (red). (H) Relative p-MLC fluorescence intensity or (I) the distance between DIC cell centroid and p-MLC fluorescence centroid at basal level (white bars) or after fMLP stimulation (black bars). Representative images obtained from at least 3 independent experiments are shown. * *P<0.05* compared to the basal level; # *P<0.05,* compared to DMSO-treated cells; ** *P<0.05,* compared to cells treated with Y27632 alone. Scale ba = (A) and (G) 5 µm; (D) 10 µm.

Neutrophil morphological polarity is maintained through a balance of ‘frontness’ and ‘backness’ signals which have been shown to be mediated by the Rho GTPases Rac/Cdc42 GTPases and RhoA, respectively [2]. The putative roles of the Rho GTPases and PAK in leading edge dynamics led us to hypothesize that PAKs may maintain neutrophil ‘frontness’ by modulating ‘backness’ during neutrophil polarization. To test this hypothesis, we assessed the effects of PAK inhibition on RhoA GTPase activation and localization using the pharmacological PAK inhibitor, PF3758309 [12], [15], [16], which inhibited the phosphorylation of PAK1/2 Thr423/402 (Fig. 2A and 2B) and PAK2 Ser20 (data not shown) in fMLP-stimulated neutrophils. While neutrophils developed a morphological polarity following fMLP stimulation, characterized by a significant increase in aspect ratio (a function of the largest cell diameter and the smallest diameter), PF3758309-treated cells underwent polarization in the absence of any stimulation (Fig. 2C). In vehicle-treated cells, fMLP-induced polarization was associated with an increase in the immunofluorescence of active RhoA-GTP (Fig. 2D and 2E) and a decrease in the colocalization of RhoA-GTP with actin (Fig. 2D and 2F), suggesting that fMLP induced active RhoA accumulation at the uropod. In contrast, PF3758309-treated cells displayed similar signatures of cell polarization in the absence of fMLP with a significantly higher level of active RhoA as compared to control cells (Fig. 2D–F). Stimulation of PAK-inhibited cells with fMLP resulted in a comparable level of active RhoA to fMLP-stimulated control cells (Fig. 2D–F). These results suggest that PAK contributes to neutrophil cytoskeletal dynamics under basal conditions by suppressing RhoA-GTP accumulation and relocalization.

Active RhoA binds to and activates a number of downstream effectors including the Rho-associated kinase ROCK [2]. The RhoA-ROCK axis has been shown to be essential for myosin II-mediated actomyosin contraction in various cell types [27] and for surface detachment of migrating leukocytes [34], in which ROCK phosphorylates myosin light chain (MLC) at Ser19 to promote the actin-stimulated ATPase activity of myosin. In neutrophils, MLC phosphorylation plays a key role in uropod retraction [35], and in the maintenance of backness polarity [36]. To test whether PAK activity has a role in actomyosin contraction, we next examined the subcellular distribution of phospho-MLC under basal and PAK-inhibited conditions. fMLP induced an increase in phospho-MLC (Fig. 2G and 2H) at sites distant from cell centroids (Fig. 2I), suggesting a polarized mechanism of actomyosin contraction. Phospho-MLC immunofluorescence was abrogated in the presence of the ROCK inhibitor, Y27632 (Fig. 2G and H). Inhibition of PAK with PF3758309 led to an accumulation of phospho-MLC in unstimulated and stimulated cells (Fig. 2G and 2H). Interestingly, the loss of MLC phosphorylation resultant from Y27632 was partially recovered by co-treatment of PF3758309 with Y27632 (Fig. 2G and 2H). Taken together, this data suggests that PAK regulates actomyosin contraction in migrating neutrophils via ROCK-dependent and-independent pathways.

### PAK Negatively Regulates focal Complex Assembly and Surface Adhesion

**Figure 3:**
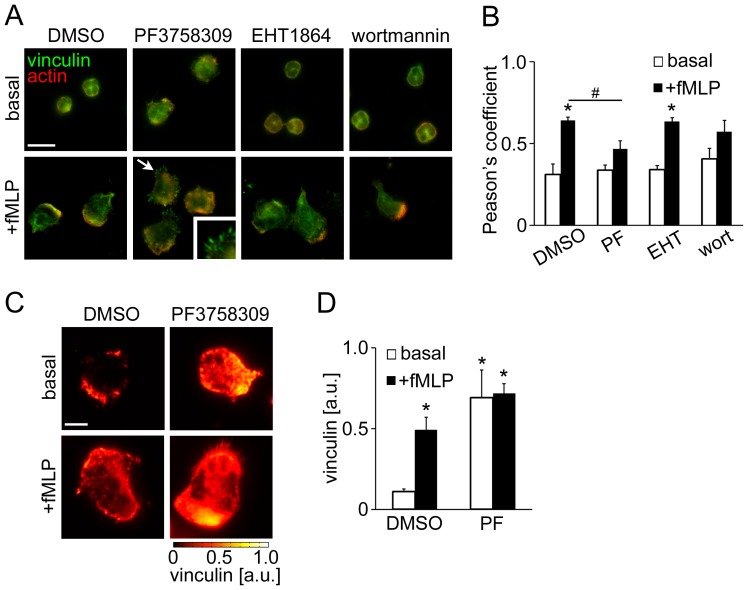
PAK inhibition enhances vinculin-mediated surface contacts. Human neutrophils adherent on fibronectin surfaces were treated in the presence or absence of fMLP (10 nM) for 3 min. In selected experiments, neutrophils were pretreated with PF3758309 (PF; PAK inhibitor, 10 µM), EHT1864 (Rac1/2 inhibitor, 50 µM) or wortmannin (PI3K inhibitor, 100 nM). (A) Cells were stained for vinculin (green) and F-actin (red). Arrows indicate filopodia-like vinculin clusters shown in the insets. (B) Pearson’s coefficient for vinculin and actin immunofluorescence in cells treated as indicated in (A). (C) Neutrophil surface vinculin was visualized using TIRF microscopy. Arbitrary units (a.u.) for vinculin signal intensity are shown. (D) The mean TIRF signals of vinculin immunofluorescence were quantified from at least 5 cells per treatment. * *P<0.05* compared to the basal vinculin signal in DMSO-treated cells. Scale ba = (A) 10 µm; (C) 2 µm.

To stabilize the lamellipodium, small adhesion structures, known as focal complexes, are formed at the leading edge via Rac/Cdc42 activity which link integrins to surface and cytoskeletal proteins [37]. To determine the contribution of PAK activity to focal complex assembly in neutrophils, we examined the localization of vinculin, a marker of focal complex assembly, under control and pharmacologically-inhibited conditions. As seen in Fig. 3A and B, vinculin localized to the cytosol of unstimulated neutrophils and accumulated at the actin-rich leading edge as well as the uropod after fMLP stimulation, indicating the presence of vinculin-containing cell adhesions. Neutrophils treated with the Rac1/2 inhibitor EHT1864 displayed a similar pattern of vinculin localization as compared to control cells, whereas treatment with the PI3K inhibitor wortmannin led to the less polarized localization of vinculin immunofluorescence (Fig. 3A and 3B). Interestingly, PAK-inhibited neutrophils formed vinculin-positive, filopodia-like structures at the cell periphery in response to fMLP (Fig. 3A). Similar vinculin clusters have been found in neutrophils treated with TNF-α [38], which confers inhibitory signals to neutrophil motility by promoting firm adhesion and limiting polarization. Accordingly, we next aimed to examine whether PAK inhibition enhanced neutrophil adhesion strength by increasing surface contacts. Neutrophil adhesion complexes were studied by total internal reflection fluorescence (TIRF) microscopy, by which immunofluorescence signals adjacent (∼150 nm) to the interface between a surface and a specimen are exclusively detected with a high spatial resolution as an indicator of surface contact area. As seen in Fig. 3C and 3D, TIRF microscopy for vinculin immunofluorescence revealed that PF3758309-treatment dramatically enhanced the level of vinculin-mediated surface contact as compared to vehicle, both in the presence and absence of fMLP. Together, these results demonstrate that PAK may negatively regulate surface adhesion during neutrophil polarization.

### PAK Plays a Role in Neutrophil Directional Migration and Intracellular Calcium Release

**Figure 4:**
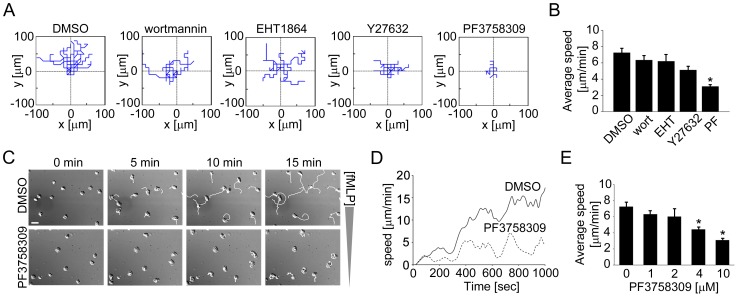
PAK inhibiton blocks neutrophil chemotaxis. Human neutrophils were treated with vehicle (0.1% DMSO), EHT1864 (Rac1/2 inhibitor, 50 µM), wortmannin (PI3K inhibitor, 100 nM), Y27632 (ROCK inhibitor, 10 µM), or PF3758309 (PAK inhibitor, 10 µM) for 15 min and chemotaxis was induced by adding fMLP (10 nM) in the outer well of an Insall chamber. Time-lapse images were obtained every 10 s for 20 min at 40× magnification using DIC microscopy. (A) The migration paths of individual cells were quantified as spider plots using MtrackJ (Image J) and MATLAB. (B) Average migration speeds of neutrophils treated as indicated in (A). (C) Representative time-lapse images of DMSO- or PF3758309-treated cells at the first 0, 5, 10, 15 min with cell paths (white). (D) Representative kinetics of cell migration was plotted against time. (E) A dose-dependent inhibitory effect of PF3758309 on average migration speed. Results were obtained from 3 independent experiments and data quantification was performed for at least 30 cells per treatment. * *P<0.05* compared to the average migration speed of DMSO-treated cells. Scale bar = 20 µm.

The above described PAK-mediated regulation of actomyosin contractility and surface adhesion led us to further examine the role of PAK in neutrophil chemotaxis. The determining components of efficient chemotaxis include directional sensing, morphological polarization and motility. Our data show that upon exposure to an fMLP gradient, neutrophils first uniformly spread and then immediately developed morphological polarity, in which the leading edge formed distinct lamellipodia to differentiate from the uropod (Fig. 4C and Movie S1). Within 20 min, neutrophils migrated toward the fMLP gradient at the average speed of 7.2±0.5 µm/min (Fig. 4A and 4B).

To better understand the roles of PI3K, Rho GTPases and PAK signaling pathways downstream of fMLP that regulate neutrophil chemotaxis, we characterized the directionality, polarity and motility of migrating neutrophils in the presence of pharmacological inhibitors. Neutrophil directionality was partially disrupted in the presence of the PI3K inhibitor, wortmannin (Fig. 4A). Although migration speed was unaffected (6.2±0.85 µm/min; Fig. 4B), wortmannin-treated neutrophils displayed impaired formation of a distinct leading edge and uropod (data not shown). The presence of the Rac1/2 inhibitor, EHT1864, led to a random migration pattern with a migration speed comparable to vehicle-treated cells (6.3±0.56 µm/min; Fig. 4A and 4B). Treatment of neutrophils with the ROCK inhibitor, Y27632, resulted in the formation of longer or undetached uropods (data not shown), although neutrophil motility was not significantly reduced (5.1±0.47 µm/min; Fig. 4A and 4B). In contrast, neutrophils treated with PF3758309 underwent enhanced spreading in response to fMLP (Fig. 4C and Movie S2) and randomly migrated at a dramatically reduced migration speed (3.1±0.21 µm/min; Fig. 4A, 4B and 4D) in a concentration-dependent manner (Fig. 4E). These results suggest that PAK activity plays a crucial role in coordinating directional sensing, polarity and motility of chemotaxing neutrophils.

**Figure 5:**
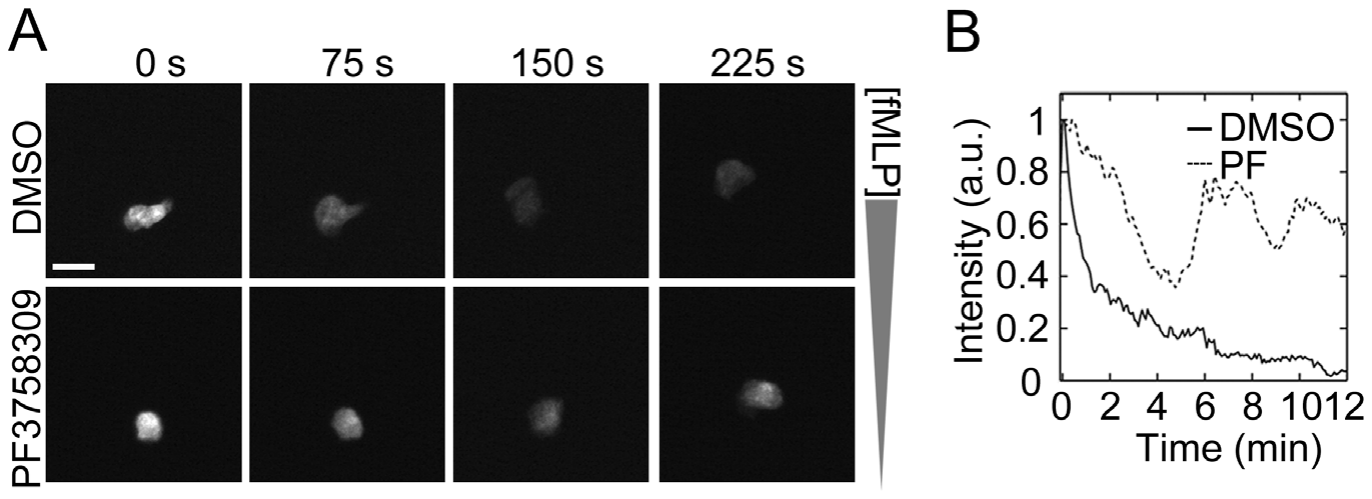
PAK inhibition alters neutrophil calcium signaling. Human neutrophils were loaded with the intracellular Ca^2+^ dye fluo-4 (2 µM) and plated on fibronectin-coated surfaces. After treatment with DMSO or PF3758309 (10 µM), neutrophil chemotaxis was induced by the addition of fMLP in an Insall chamber. Intracellular Ca^2+^ release was monitored every 5 s for 15 min by fluorescent microscopy. (A) Time-lapse images after the peak Ca^2+^ spike were shown. (B) Ca^2+^ spikes in cells treated with 0.1% DMSO (black line) or PF3758309 (PF; PAK inhibitor, dotted line) were quantified and presented as mean intensity of at least 5 neutrophils in a field of view. Data shown is representative from 3 independent experiments. Scale ba = 20 µm.

fMLP receptor signaling induces intracellular Ca^2+^ transients via the activation of the Gβγ complex and phospholipase C, triggering Ca^2+^-dependent downstream pathways such as protein kinase C [2], [39]. These pathways have been shown to collectively play a role in cytoskeletal dynamics in neutrophils [40]. PAK1-deficient bone marrow-derived mast cells have been shown to exhibit diminished intracellular calcium mobilization [9], [13]. However, a role for the PAK pathway in the coupling of calcium signaling during neutrophil chemotaxis has not been explored. Next, to characterize the role of PAK in intracellular Ca^2+^ mobilization, neutrophils were loaded with the Ca^2+^-sensitive reporter dye fluo-4 AM and allowed to migrate under a fMLP gradient while intracellular Ca^2+^ release was measured for 15 min. As shown in Fig. 5, neutrophils exhibited a single spike of Ca^2+^ release upon exposure to fMLP, followed by a gradually decreasing level of Ca^2+^ during chemotaxis. In contrast, neutrophils treated with the PAK inhibitor, PF3758309, displayed multiple peaks of Ca^2+^ release and failed to develop polarized morphology or migrate toward the fMLP gradient (Fig. 5). These data imply that PAK activity contributes to the regulation of intracellular Ca^2+^ transients during neutrophil chemotaxis.

## Discussion

**Figure 6:**
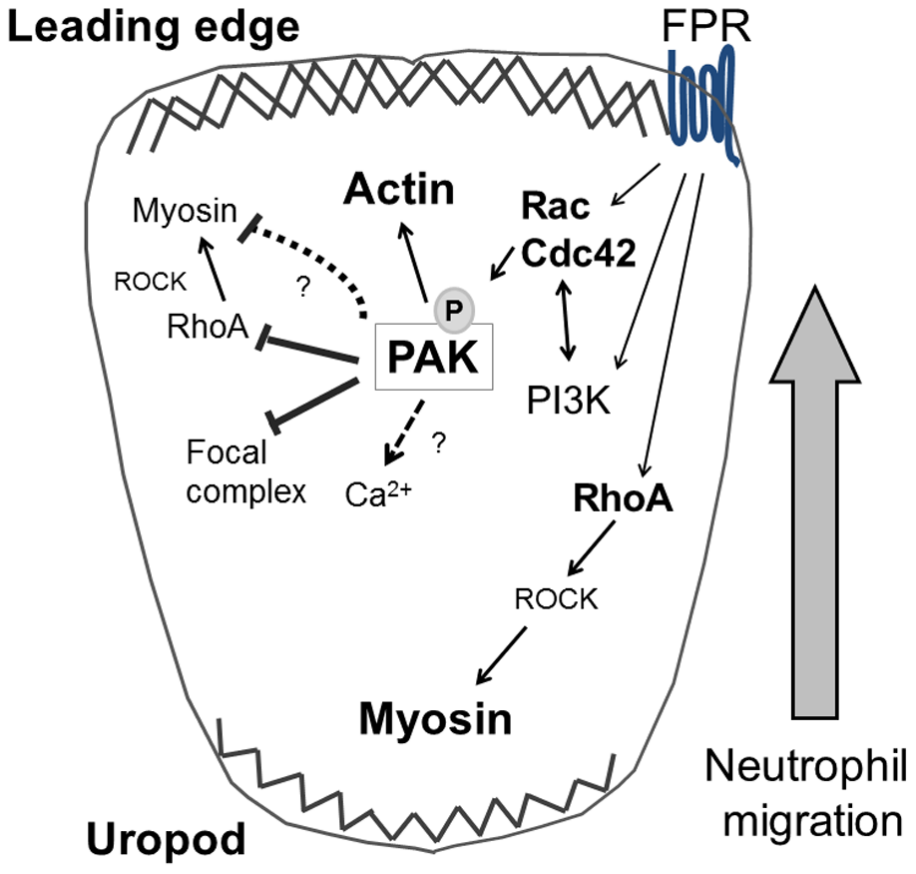
A model for PAK-mediated cytoskeletal regulation in human neutrophils. PAK coordinates the crosstalk between Rho GTPases and cytoskeletal dynamics in migrating neutrophils.

The efficient migratory ability of neutrophils requires a network of interactions between distinct cytoskeletal signaling systems, namely frontness signals regulated by actin-dependent membrane protrusion and backness signals mediated by myosin-dependent cell contraction. Recent studies suggest that crosstalk processes between frontness and backness modules during the initiation, establishment and maintenance of neutrophil polarization are dynamic and complex [41]. Moreover, the mediators of temporal and spatial regulation in this crosstalk network remain to be elucidated. In this study, we identified the p21-activated kinase PAK as a regulator of neutrophil cytoskeletal reorganization. The PAK family proteins serve as effectors downstream of Rac and Cdc42 GTPases and are noted for roles in cytoskeletal dynamics, gene transcription, survival signaling, and cell cycle progression [25], [23]. Here we find that the kinase activity of PAK is required for the efficient directional migration of human neutrophils, where PAK2 may serve key roles in frontness signals at the leading edge. The absence of PAK activity resulted in an impairment of polarization, which was associated with increased backness signals and surface adhesions. Together, as depicted in Fig. 6, our data describe a differential spatial regulation of PAK isoforms in fMLP-stimulated human neutrophils and highlights a potential mechanism for PAK-mediated crosstalk between Rho GTPases during cytoskeletal reorganization.

The expression of the six PAK isoforms differs according to cell and tissue type, implying that PAK family isoforms may have specific and distinct functions in differing physiological contexts [20], [21]. Recent studies have reported functional differences for PAK1 and PAK2 in various model systems. In hematopoietic stem and progenitor cells, PAK2 plays a major role in coordinating cytoskeletal and proliferative pathways that are essential for engraftment of these cells [14]. PAK1 and PAK2 have been shown to differentially modulate adhesion, RhoA activity and MLC phosphorylation during mast cell degranulation [13] and tumor cell migration [42]. Here, we find that human neutrophils express PAK1, PAK2 and PAK4, and that PAK2 may play a specific role in the regulation of actin dynamics at the leading edge upon neutrophil activation. Whether PAK1 and PAK4 have any role in mediating cytoskeletal regulation at non-leading edge regions of migrating neutrophil requires further investigation. Along these lines, PAK1, but not PAK2, has been shown to positively regulate IgE-mediated degranulation in mast cells by regulating extracellular Ca^2+^ influx through a mechanism involving cytoskeletal dynamics, although the molecular details of such events remain to be explored [9], [13]. It is possible that the dysregulation of Ca^2+^ mobilization by PAK inhibition in our human neutrophil study was a direct result of the blockade of the activity of specific PAK isoforms. Further studies are required to determine whether any of these PAK isoforms can serve as a scaffold for signaling proteins to facilitate polarization, dependently or independently of their kinase activity, in human neutrophils. Notably, the pharmacological PAK inhibitor PF3758309 utilized in this study does not allow for the selective inhibition of specific PAK isoforms, as this compound inhibits all PAK isoforms (PAK1–6) [12]. Future studies will be aim to define specific roles for PAK isoforms in neutrophil function using bone-marrow specific, inducible PAK-deficient mouse models, together with second generation PAK inhibitors with distinct target specificities.

Antagonistic signals between Rac1/2, Cdc42 and RhoA stabilize the polarity of migrating cells by restricting actin polymerization to the leading edge and myosin-dependent contraction to the uropod [2], [43]. For efficient uropod retraction during migration, the RhoA-ROCK-myosin axis plays an essential role in myeloid cell lines, as the expression of dominant negative RhoA [36] or the pharmacological inhibition of ROCK [36], [44] leads to multiple lamellipodial protrusions and a mislocalization of phosphorylated MLC. In our experiments, the inhibition of PAK in unstimulated neutrophils promoted the accumulation of active RhoA and phospho-MLC at the cell periphery (Fig. 2), suggesting that the basal PAK activity can suppress the development of backness signals. The molecular mechanisms that bridge PAK activation and RhoA regulation in neutrophils have not been completely described. As Rho GTPases can be activated by multiple GEFs, which promote the release of GDP and the binding of GTP in specific cellular contexts [27], it is possible that PAK may modulate the activity of Rho GEFs such as GEFH1 [45] and PDZ RhoGEF [46] in a localized manner. Furthermore, our data imply that PAK can regulate MLC phosphorylation through RhoA/ROCK-independent pathways (Fig. 2G and 2H). Potential alternative pathways for MLC phosphorylation include Ca^2+^-dependent myosin light chain kinase (MLCK), as the phosphorylation of MLCK by PAK has been shown to inhibit MLCK activity [47].

In motile cells, adhesion strength is one of the determining factors for migratory ability. Focal complexes, consisting of integrins and adhesion complex proteins such as vinculin, stabilize the lamellipodium by supporting surface attachment, and contribute to efficient cell migration [37]. Several studies have shown that in transfected cell lines, the binding of active Rac/Cdc42 to PAK induces the recruitment of PAK to focal complexes [48], [49]. A study by Delorme-Walker et al. utilizing an epithelial cell line demonstrated that PAK inhibition resulted in the disruption of actin turnover, myosin II and focal adhesion dynamics [50]. In rapidly migrating cells such as neutrophils, however, the formation of focal complexes is less visible, likely as a result of their rapid migration [37]. Our current study demonstrated that the absence of PAK activity led to a filopodia-like clustering of vinculin in human neutrophils (Fig. 3). These results provide a novel regulatory mechanism for PAK-dependent surface adhesion in migrating neutrophils.

In summary, our data describe PAK as a key coordinator of cytoskeletal dynamics during cytoskeletal reorganization in human neutrophils and demonstrates that PAK mediates the crosstalk between the Rho GTPases to maintain the balance of front and back polarity signals. Future studies are required to characterize the functional roles of PAK in neutrophil inflammatory responses such as oxidative burst, degranulation, phagocytosis and neutrophil extracellular trap formation.

## Supporting Information

Movie S1 (MOV)

Movie S2 (MOV)
